# Sued, Subpoenaed or Sworn in: Use of a Flipped-Classroom Style Medicolegal Workshop for Emergency Medicine Residents

**DOI:** 10.5811/westjem.17809

**Published:** 2024-06-14

**Authors:** Kathleen S. Williams, Tatiana Griffith, Sean Gaynor, Thomas Johnson, Alisa Hayes

**Affiliations:** * Medical College of Wisconsin, Department of Emergency Medicine, Milwaukee, Wisconsin; † Healthpartners Regions Hospital Emergency Medicine, St. Paul, Minnesota; ‡ Leib Knott Gaynor LLC, Milwaukee, Wisconsin

## Abstract

**Background:**

It is an unfortunate truth that Emergency Medicine (EM) physicians will, at some point, have contact with the medicolegal system. However, most EM residency training programs lack education on the legal system in their curriculum, leaving EM physicians unprepared for litigation. To fill this gap, we designed a high-yield and succinct medical legal workshop highlighting legal issues commonly encountered by EM physicians. We aimed to determine the effectiveness of this curriculum by measuring pre and post knowledge questions.

**Methods:**

A two-hour session included a case-based discussion of common misconceptions held by physicians about the legal system, proper steps when interacting with the legal system and review of legal documents. This session was developed with the involvement of our hospital legal counsel and discussed real encounters. The effectiveness of the session was determined using pre- and post-session surveys assessing participant knowledge and comfort approaching the scenarios.

**Results:**

A total of 34 EM residents had the opportunity to complete this workshop as a part of their conference curriculum. A total of 26 participants completed the pre-survey and 19 participants completed the post-survey. No participants had previous training in the legal aspects of medicine, including handling a subpoena, serving as a witness, or giving a deposition.

The pre-survey demonstrated that there was significant uncertainty surrounding the processes, definitions, and the legal system interaction. Many participants stated they would not know what to do if they received a subpoena (85.71%), were called as a witness in a trial (96.43%) or receive correspondence from a lawyer (96.43%).

The post survey revealed an increased knowledge base and confidence following the session. 100% of residents reported knowing what to do after receiving a subpoena, being called as a witness and understanding the process involved in giving a deposition. All residents reported that the session was beneficial and provided crucial information.

**Conclusion:**

EM residents have limited baseline understanding of how to approach common legal scenarios. Educational materials available for this curriculum topic are limited. Based on the rapid knowledge increase observed in our residents, we believe our workshop could be adapted for use at other residency programs.

## BACKGROUND

Emergency physicians (EP) are at the frontline of acute care and as a result have frequent interactions with the United States medicolegal system. Physicians in specialties considered “high risk,” including emergency medicine (EM), experience a higher rate of malpractice claims than average, with 99% of physicians in these specialties experiencing malpractice litigation by the age of 65.^1^ However, only 18% of EM residency programs devote more than four hours per year of curriculum time to medicolegal topics, including items such as medical malpractice and risk management education.^2^ In a survey of Australian EPs, 41% of respondents reported receiving training in this area. Also, while 71% had attended court as an expert witnesses, only 23% considered themselves skilled in participating in courtroom trials.^3^

While similar data does not exist evaluating the preparation of EPs in the US, previous studies support that lack of medicolegal education impairs a physician’s ability to assist the court and form an accurate opinion.^3^ A solid educational foundation in the legal aspects of medicine is especially important for EPs, who often interact with patient populations requiring interaction with the medicolegal system. These situations include abuse, assault, domestic or gun violence, other traumatic injury, and forensic toxicology. Historically, training focused on this area happened in real time, with few institutions implementing forensic medicine training to better address their patients’ forensic needs.^4^

Much of the current literature pertaining to medicolegal education is from countries outside the US. In an Australian EM training program, a six-month forensic medicine rotation improved the technical, assessment, and clinical skills of their EM residents.^5^ In the US, few residency programs have implemented direct simulations of trial scenarios, educational lectures, and case-based discussions to improve their residents’ ability to interact with the legal system. These programs historically have consumed substantial time, with a range of six hours to several months in duration. Partnerships with local law schools have allowed EM residents to receive hands-on experience with malpractice litigation and have been shown to improve their confidence in navigating the legal system.^6^^,^^7^ The American Board of Emergency Medicine has included understanding legal concepts in its “Model of the Clinical Practice of Emergency Medicine.”^8^ However, it is still to be determined how best to cover these topics as part of the EM training curriculum.

## OBJECTIVES

We aimed to determine whether a two-hour, case-based curriculum developed with our hospital legal counsel would efficiently improve our residents’ comfort with approaching three common legal scenarios encountered by EPs and strengthen resident understanding of their own rights within the medicolegal system.

## CURRICULAR DESIGN

After repeated instances of our residents’ receiving subpoenas, we reached out to our hospital legal counsel regarding the need to develop a curriculum focused on common scenarios encountered by EPs. A short, 30-minute, didactic review was developed, and three case scenarios were introduced. Our legal counsel was able to modify actual documents and forms that had been sent to physicians whom he had previously represented and use them to create small-group discussions surrounding how to best approach these scenarios (see Supplement). Topics covered by the three case scenarios included responding to a subpoena, serving as a witness, and being involved in a deposition. This session was held in August 2021.

The learners were given time to review the documents and answer discussion questions regarding the case as a small group. They then returned to the larger group to review their findings and receive feedback from EM faculty and our legal counsel regarding their conclusions.

## IMPACT/EFFECTIVENESS

Prior to the beginning of the workshop, participants were asked to complete an anonymous, voluntary survey. Residents were asked to complete an identical survey immediately following the completion of the workshop. The survey included nine multiple-choice questions aimed at evaluating the residents’ baseline medicolegal knowledge and five questions assessing trainee comfort with each topic highlighted in the session, using a Likert scale. Examples of knowledge questions included the following: “The differences between being deposed and testifying in court are____”; and “If I am subpoenaed to testify for a patient I saw, what should my next step be?” Approval for this study was obtained from the Quality Improvement/Quality Assessment Review Committee of the Department of Emergency Medicine at the Medical College of Wisconsin and was deemed institutional review board-exempt.

A total of 34 postgraduate year 1–3 EM residents had the opportunity to complete this workshop as a part of their weekly conference curriculum. The pre-survey was started by 29 participants with 26 completing all questions. The post-survey was completed by 19 participants. All the participants stated that they did not have previous training in the legal aspects of medicine, including handling a subpoena, being called as a witness, or giving a deposition. Postgraduate year of training was not asked on the survey to avoid identification of the participants, given the small sample size.

**Figure 1.**
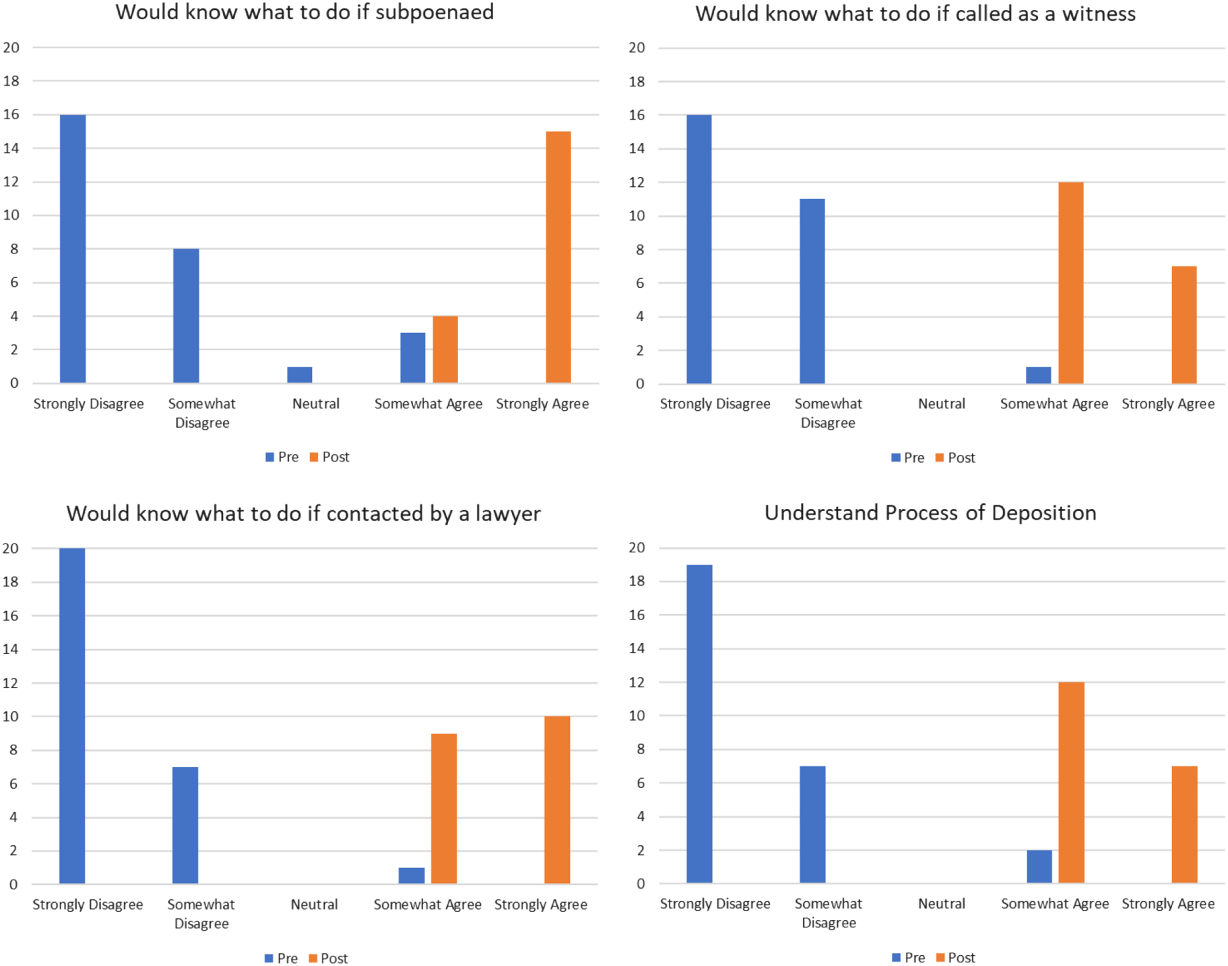
Pre- and post-survey scenario results demonstrate improvement in self-reported knowledge of emergency residents on how to approach several common legal scenarios.

The pre-survey demonstrated there was significant uncertainty surrounding the processes, definitions, and intentions of the legal system (Figure 1). A large majority of participants stated they would not know what to do if they received a subpoena (85.71%), were called as a witness in a trial (96.43%), or received correspondence from a lawyer (96.43%). Responses revealed uncertainty with the goal of deposition and how it differed from trial, with only 40.74% of residents indicating that practice for trial was not an included goal and 56.26% knowing that only one person is being questioned during deposition.

Residents left the workshop with a deeper understanding of their legal rights and the proper steps to take when contacted regarding litigation. On the post-survey, 100% of residents reported knowing what to do after receiving a subpoena, being called as a witness for a trial, and understanding the process involved in giving a deposition, and 94.74% agreed that they were aware of the policy statements by the American College of Emergency Physicians surrounding acting as an expert witness. When the session was evaluated overall, 100% “strongly agreed” the session was helpful. These pre- and post-session changes in self-assessment of knowledge (questions noted in Figure 1) were found to be statistically significant (*P* < 0.05) when a chi-squared analysis was performed.

**Figure 2.**
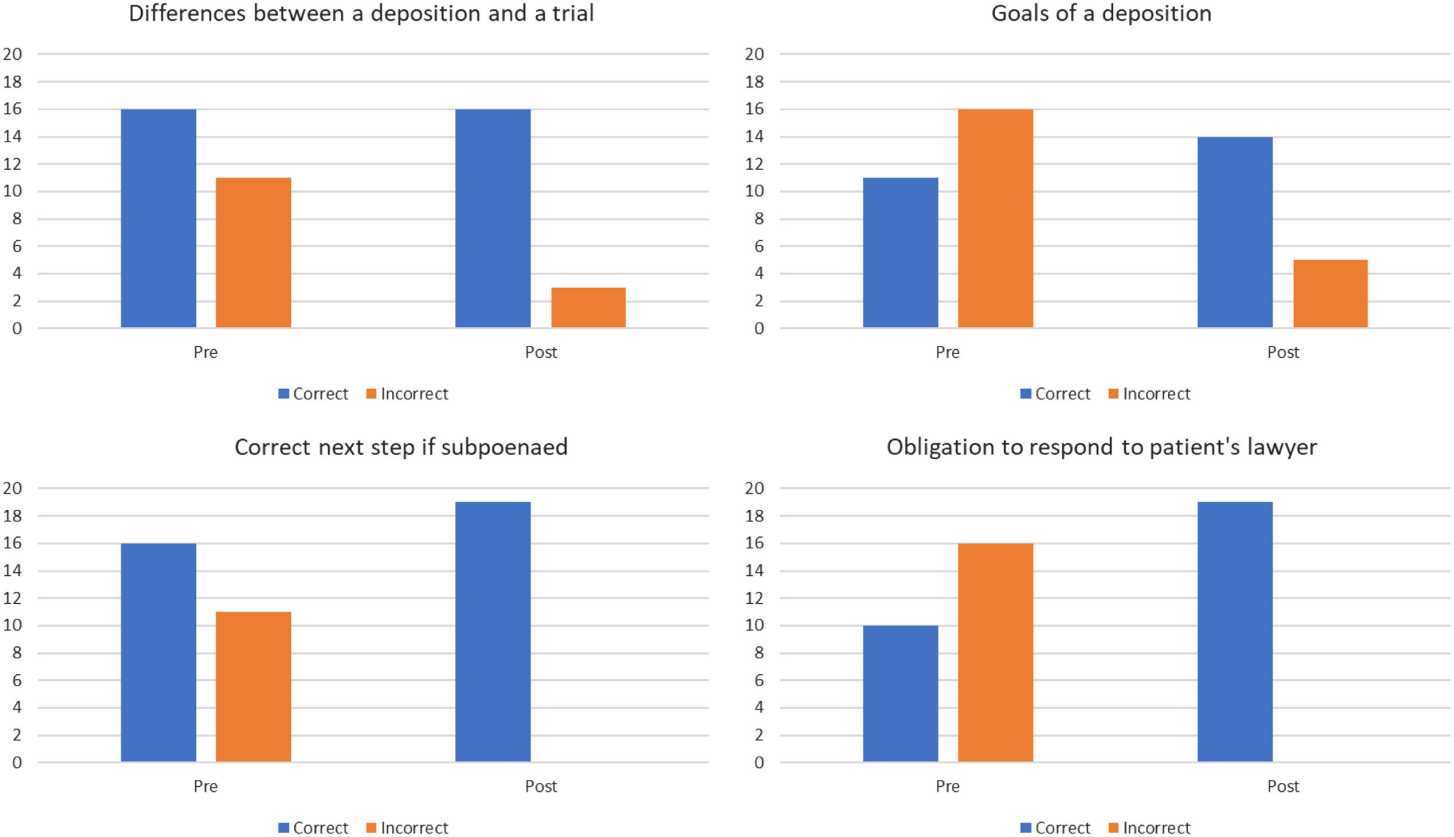
Knowledge-based questions assessed before and after the session demonstrate an increase in correct response, although the majority were not statistically significant.

Regarding knowledge related to the goal of a deposition, differences between a deposition and a trial, obligations to respond to a lawyer, residents’ correct-response rate improved after the session (Figure 2). These differences were not found to be statistically significant. However, when we performed a chi-squared analysis we found a statistically significant improvement in knowledge related to being contacted by a lawyer (*P* < 0.05).

At the end of the workshop, there was a distinct shift from residents lacking a basic understanding of the medicolegal system, or what role physicians serve, to being well prepared for the deposition process and how to properly respond to a legal correspondence. Residents were provided with the framework required to navigate litigation and provided a space to discuss common medicolegal scenarios that EPs face. These results were achieved in a relatively brief time frame, which indicates that short, case-based scenarios can be implemented to effectively improve resident knowledge and provide them with information that can be immediately applied. Based on the success of this workshop, we believe that similar medicolegal sessions could be adapted for other residency programs to reduce the gap between experience and education.

Our workshop model did have limitations including limited sample size and utilization of a single training site. Because each state within the US has its own legal nuances, no legal curriculum can be universally applied to all residency programs. Additionally, we observed a 27% drop in participation on the post-survey when compared to the pre-survey. Sustainability of the impact we observed has not yet been assessed in a delayed fashion.

Moving forward, integrating methods used by other programs, including expanding to multiple sessions, leveraging partnerships with local law schools, using mock trial scenarios, or creating forensic science electives, may further bolster this curriculum. We identify that the number of topics covered in this curriculum are limited. Certainly, additional work can be done to further expand this basic legal education to cover the scenarios EPs routinely encounter.

## CONCLUSION

Based on the current literature and the experiences of our residents, EM trainees are unprepared for their encounters with the legal system and require more education on this topic. Given the frequent contact that emergency physicians have with the medicolegal system, further work is essential to improve trainee preparedness for contact with the legal system. There remains a vast opportunity for this area of resident education to further grow and develop. Medical educators within EM should continue to explore how to best cover these topics within their own programs.

## Supplementary Information




