# Supplementary material for: Sued, Subpoenaed or Sworn in: Use of a Flipped-Classroom Style Medicolegal Workshop for Emergency Medicine Residents
Source: West J Emerg Med. 2024 Jun 14;25(4):579–83. doi: 10.5811/westjem.17809 (PMC11254144; doi:10.5811/westjem.17809)
Supplement: Supplementary file 1 [file wjem-25-579-s001.pdf]

MOCK CRIMINAL CASE SUBPOENA

On January 10, 2021, you treated Linda Carter, a 48 year-old female patient who presented to the ER with extreme right shoulder pain. She presented with her husband, Mel, and claimed that she had tripped at home and landed squarely on her shoulder. She was very tearful and in significant pain. You ordered various imaging studies which revealed the possibility of a right rotator cuff tear. Additionally, you contacted orthopedics who provided instructions and medications for discharge with directions to follow-up at their clinic the following day for a consult. Your note of this visit does not document anything nefarious, outlines her general description of the fall and merely comments that her husband was present. Additionally, the note documents that "patient and husband had no questions and understood discharge and follow-up care instructions."

Recently, you were unexpectedly served with the attached subpoena from the local DA to appear at a trial against Ms. Carter's husband, Mel. You do not have an independent recollection of Ms. Carter and a review of your records provided some faint recollection of the patient and her husband, mainly that they were both fairly quiet. You contacted the DA and spoke with a legal assistant who gave you some startling background. Approximately one month after your treatment, police were called to the Carter home for a domestic disturbance and they found Linda severely beaten and bruised. During her hospitalization, at a different facility, imaging revealed an unhealed rotator cuff. The police officers questioned Ms. Carter and she detailed a lifetime of abuse at the hands of her husband. She also revealed that her rotator cuff injury was caused by her husband chasing after her and pushing her violently to the floor. Mel has a history of five prior minor misdemeanor domestic violence cases all of which were hampered by his wife's unwillingness to cooperate. Now, Linda has had enough and the DA is ready to put Mel behind bars. The legal assistant has indicated they want you to testify that the rotator cuff injury was likely caused by Mel pushing his wife to the floor. She indicated "not to worry" and that "you can talk to the DA the day of the trial."

State of Wisconsin | **Circuit Court** | **Great Lakes County**

STATE OF WISCONSIN

Plaintiff,

-VS-

Mel Carter

Defendant,

DA Case No.: 2021XF232
Assigned DA/ADA: John Smith

Court Case No.: 2021CF44234

SUBPOENA

The State of Wisconsin to:

John/Jane Doe, M.D.
Medical College of Wisconsin – ER

CERTIFICATE OF SERVICE

Date served: August 10, 2021
Time served: 8:58 a.m.
Serving Agency: Great Lakes Sheriff's Department
Served By: Officer Friendly
Date Received: August 10, 2021
Witness: Mike Jones
Witness Address: ER Department

(if other than address on document)

YOU ARE REQUIRED TO APPEAR AND TO GIVE EVIDENCE:

APPEARANCE INFORMATION

Jury Trial on Tuesday, September 15, 2021 at 8:15 AM in C-6, in front of the Honorable Judge Jim Roberts

Comments: **Patient: Linda Carter**
Date of Treatment: January 10, 2021

Please plan to arrive at the District Attorney's office 15 minutes prior to the time indicated for this proceeding. This will give you an opportunity to discuss your testimony with Attorney Smith, who is handling this case. The District Attorney's office is located at Great Lakes County Courthouse, Room 20A. Please bring the copy of your subpoena with you in order to be paid your witness fee. **If you have any questions about this subpoena, please contact the Victim Witness Program at 920-832-5024.**

You must telephone (920) 832-4732 between 5:00 PM on the date prior to the hearing and 8:00 AM on the day of the hearing for a tape recorded message to be advised if this hearing has been cancelled. Failure to do so will result in denial of witness fees to witnesses appearing for cancelled proceedings.
Failure to appear may result in punishment for contempt, which may include monetary penalties, imprisonment and other sanctions.

ISSUING OFFICIAL:

Robert Phillips
District Attorney
