# Supplementary material for: Sued, Subpoenaed or Sworn in: Use of a Flipped-Classroom Style Medicolegal Workshop for Emergency Medicine Residents
Source: West J Emerg Med. 2024 Jun 14;25(4):579–83. doi: 10.5811/westjem.17809 (PMC11254144; doi:10.5811/westjem.17809)
Supplement: Supplementary file 2 [file wjem-25-579-s002.pdf]

August 10, 2021

Brian King, Esq.
414.888-8888

Dr. Jane/John Doe
ER Department - MCW

Re: **My Clients**: The Estate of Mike Richards/Elsa Richards, Gabriel and Alexa Richards.
Date of Treatment: May 10, 2020

Dear Dr. Doe:

I apologize for the intrusion. I represent The Estate of Mike Richards, his wife, Elsa, and their two children, Gabriel and Alexa. We are exploring possible legal action pertaining to his untimely passing on May 12, 2020. As you may recall, Mike was a young, thirty-eight-year old gentleman that you treated in the ER on May 10, 2020. Mike had undergone a cholecystectomy earlier in the day on May 10, 2020 at an outside facility, Great Lakes Medical Center. He presented to the ER that evening with complaints of severe abdominal pain with guarding which he described as 10 out of 10, low-grade fever, increased respirations, tachycardia and an overall general feeling of malaise. If you recall, you ordered blood work which showed an elevated white blood cell count with a left shift and an abdominal x-ray which, although not definitive, revealed possible small amounts of gas within the peritoneal cavity. You obtained a surgical consult and indicated a diagnosis of "possible bowel perforation secondary to cholecystectomy."

Although Mike was transferred to the general surgery unit, he did not undergo surgery immediately and not until May 12th, at which time his bowel was totally and completely necrotic and unsalvageable. He was severely septic and died shortly thereafter. He left behind his wife of 16 years and their two children, ages 8 and 6. In an effort to explore what occurred and have a better understanding of his treatment, I respectfully request you fill out the attached questionnaire. I want to assure you that my clients know you did all that you could for them, but the lack of information being provided regarding this incident is causing them extreme grief and emotional distress. Please note we are not looking to sue you but asking you for some just brief insight to help alleviate some of the pain this grieving family is experiencing.

If you have any questions, feel free to call me. Otherwise, you can just send the questionnaire back to me in the self-addressed, postage paid envelope. It will be for my eyes only. Again, thank you deeply on behalf of my clients.

Very truly yours,

Brain King

Brian King, Esq.
Enclosure

MEDICAL QUESTIONNARE

Please circle the best answer and feel free to add any explanation if necessary.

1.	A potential bowel perforation is a medical emergency?	Yes	No
2.	A cholecystectomy should not generally cause injury to a patient's bowel?	Yes	No
3.	You believed the potential bowel perforation was caused by the cholecystectomy?	Yes	No
4.	The x-ray you ordered provided an indication that Mr. Richards' bowel was potentially perforated?	Yes	No
5.	You arrived at the opinion that Mr. Richards was septic in the ER?	Yes	No
6.	Sepsis can be a potentially life-threatening condition?	Yes	No
7.	Generally, it is better for the patient the earlier sepsis is identified and treated?	Yes	No
8.	Generally, it is better for the patient the earlier a bowel perforation is identified and treated?	Yes	No
9.	You believed Mr. Richards was facing a life threating condition?	Yes	No
10.	When Mr. Richards left your care, you believed he would live?	Yes	No
11.	As an ER physician, you believed that timely surgical intervention was likely necessary to treat Mr. Richards' condition?	Yes	No
12.	You did everything you could to get Mr. Richards timely treatment for his condition?	Yes	No

Date:_____________

____
Dr. Doe
