# Supplementary material for: Sued, Subpoenaed or Sworn in: Use of a Flipped-Classroom Style Medicolegal Workshop for Emergency Medicine Residents
Source: West J Emerg Med. 2024 Jun 14;25(4):579–83. doi: 10.5811/westjem.17809 (PMC11254144; doi:10.5811/westjem.17809)
Supplement: Supplementary file 3 [file wjem-25-579-s003.pdf]

LKG

LEIB KNOTT GAYNOR

August 10, 2021

Stan Smith, Esq.
414.888-8888

Dr. Jane/John Doe
ER Department - MCW

Re: My Client: Rita Jones
Date of Treatment: June 10, 2020

Dear Dr. Doe:

Please be advised that I represent Rita Jones, a fifty-year old women you wonderfully treated in the ER on June 10, 2020 following an automobile accident she suffered. As you may recall, Ms. Jones was taken by ambulance to the Froedtert ER after the vehicle she was driving was struck in the side by a driver making an illegal turn. Unfortunately, since her ER visit, Ms. Jones has endured significant pain and suffering and has sought recompense from the other driver and his insurer, but to no avail. As such, on Ms. Jones' behalf, I have been forced to file a lawsuit against the offending driver and his insurance provider.

Ms. Jones has spoken to me kindly about the care you provided her. She has informed me that you calmed her down, spoke to her plainly and gave her reassurance. While I know you are very busy, I am writing to see if you would be willing to speak to me about the care you provided and the diagnoses you made. I do not believe the call will be very long and it would be such a favor to your patient, Ms. Jones if you would be willing to participate. You can reach me at the number above or simply send me and email and we can coordinate a time to talk. Thank you in advance for your cooperation and helping my client in a time of need.

Very truly yours,

Stan Smith

Stan Smith

LEIB KNOTT GAYNOR LLC

219 North Milwaukee Street | Suite 710 | Milwaukee, WI 53202 | 414-276-2102 | (fax) 414-276-2140

www.lkglaw.net
